# Systematic examination of the PREVENT equations for cardiovascular disease risk

**DOI:** 10.1016/j.ajpc.2026.101502

**Published:** 2026-03-13

**Authors:** Vaishnavi Krishnan, Xiaoning Huang, Chiadi E. Ndumele, Janani Rangaswami, Josef Coresh, Amanda M. Perak, Nilay S. Shah, Donald M. Lloyd-Jones, Sadiya S. Khan

**Affiliations:** a Division of Cardiology, Department of Medicine, Northwestern University Feinberg School of Medicine, Chicago, IL, USA; b Section of Preventive Medicine & Epidemiology, Department of Medicine, Boston University Chobanian & Avedisian School of Medicine, Boston, MA, USA; c Division of Cardiology, Johns Hopkins University School of Medicine, Baltimore, MD, USA; d Washington DC VA Medical Center and George Washington University School of Medicine, WA D.C, USA; e Department of Population Health, New York University Grossman School of Medicine, NY, NY, USA; f Department of Preventive Medicine, Northwestern University, Chicago, IL, USA; g Department of Pediatrics, Northwestern University, Chicago, IL, USA

**Keywords:** Cardiovascular disease risk, CKM health, Risk communication

## Abstract

**Background:**

The Predicting Risk of cardiovascular disease (CVD) EVENTs (PREVENT) equations accurately estimate CVD risk, but how different combinations of ages and risk factors translate into risk estimates, including combinations required to meet clinically meaningful risk thresholds, is not intuitive.

**Methods:**

We calculated sex-specific estimatesion of 10-year risk of total CVD (composite of atherosclerotic CVD and heart failure) for a hypothetical person between the ages of 30–79 years with the PREVENT-CVD base equations and varied individual or multiple risk factors simultaneously, with all other risk factor levels set at sex-specific, population-based average values. Next, we examined how specific age and risk factor combinations would exceed clinically-meaningful thresholds. Secondary analyses included the PREVENT-CVD equations with add-on predictors and the 30-year PREVENT-CVD equations.

**Results:**

Ten-year risk estimates for a hypothetical person with average risk factor levels ranged from 0.3 %-17.4 % for a female and 0.7 %-22.8 % for a male with the PREVENT-CVD base equations, exceeding the clinically-relevant risk threshold of ≥7.5 % at age 68 years if female and at age 63 years if male. Additionally, a hypothetical person with Stage 3 CKD and diabetes would exceed the 10-year risk threshold of ≥7.5 % with the PREVENT-CVD equations at age 43 years if female and at age 36 years if male. Similar patterns were observed with add-on predictor equations. With average risk factor levels, 30-year PREVENT-CVD risk estimates ranged from 2.5 %-20.5 % for a female and 4.8 %-26.5 % for a male.

**Conclusions:**

These results can support clinicians and patients in the interpretability of the PREVENT equations and can inform clinician-patient discussions on preventive efforts.

**Figure:**
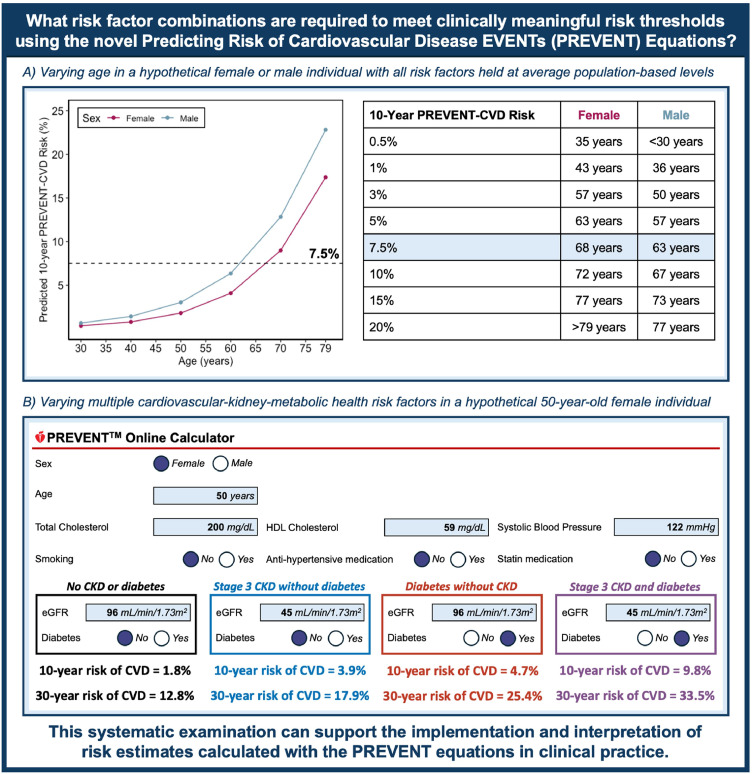
**Central Illustration: Varying clinical risk factor profiles using the American Heart Association PREVENT™ equations.***Panel A:* Ten-year PREVENT-CVD risk estimates for in a hypothetical female or male from 30 to 79 years of age with average population-based values, no diabetes or smoking, and no anti-hypertensive or statin medication, and corresponding approximate ages at which different thresholds of risk would be met. *Panel B:* Example 10-year and 30-year CVD risk calculations for varying clinical risk factor profiles (e.g., with/without diabetes, chronic kidney disease [CKD]) in a hypothetical 50-year-old female who has average risk factor levels.

## 1 Introduction

Risk assessment is broadly recommended to support clinician-patient discussions for the primary prevention of cardiovascular disease (CVD) and guide preventive strategies, such as initiation of lipid-lowering and anti-hypertensive therapies [1]. Specifically, clinical practice guidelines in the US and across the world (e.g., Europe, Australia) recommend the use of multivariable equations to quantitatively estimate absolute risk of CVD [[1], [2], [3]]. This well-established paradigm of risk-based prevention, where the intensity of the prevention effort is matched with absolute predicted risk, has been the cornerstone of preventive cardiology for more than three decades. Over this time, risk prediction equations have evolved to provide accurate and contemporary estimates by accounting for shifts in secular trends of CVD, incorporating the growing evidence base for predictors, and enabling broader outcome ascertainment. The American Heart Association Predicting Risk of CVD EVENTs (PREVENT™) equations were developed in 2023 to account for the growing burden of cardiovascular-kidney-metabolic (CKM) syndrome, included new predictors of kidney, metabolic, and social risk, and defined a broader outcome for total CVD (a composite of atherosclerotic CVD [ASCVD] and heart failure [HF]) [[4], [5], [6], [7]].

While these updates resulted in a more comprehensive, accurate, and precise approach to risk assessment, these changes also added a new complexity for interpretation of how an individual’s risk factor burden translates into their CVD risk estimate. For example, the PREVENT-CVD base equations have 19 coefficients that account for risk factors, treatment status, and age-risk factor interaction terms. The novel optional predictors in the expanded equations add up to three more coefficients for urine albumin-creatinine ratio (UACR), hemoglobin A1c (HbA1c), and social deprivation index (SDI) [4]. Consequently, risk estimates derived from these equations may not be intuitive for clinicians or patients. Examining how different combinations of clinical risk factor levels translate into CVD risk estimates can support communication among clinicians and patients, as previously conducted for prior risk models [[8], [9], [10]].

Therefore, we conducted a systematic analysis across the eligible range for each risk factor to demonstrate the intrinsic properties of the PREVENT-CVD equations and define what combinations of risk factor levels would result in clinically meaningful risk thresholds (e.g., PREVENT-CVD≥7.5 % or ≥20 %).

## 2 Methods

### 2.1 The PREVENT equations

The PREVENT equations represent a suite of models, which include total CVD (PREVENT-CVD; a composite of ASCVD and HF) and each individual CVD subtype (PREVENT-ASCVD, PREVENT-HF) [4,5]. These sex-specific equations enable prediction of 10-year risk among individuals aged 30–79 years of age and 30-year risk among individuals aged 30–59 years of age [4]. Input predictors in the PREVENT-CVD base equations include age, high-density lipoprotein cholesterol (HDL-C), total cholesterol (TC), systolic blood pressure (SBP), estimated glomerular filtration rate (eGFR), diabetes status, current smoking status, and treatment with anti-hypertensive or statin medication. Input predictors that are additionally included in the PREVENT-CVD expanded equations include urine albumin-creatinine ratio (UACR), hemoglobin A1c (HbA1c), and Social Deprivation Index (SDI). PREVENT-ASCVD includes the same input predictors as PREVENT-CVD, while PREVENT-HF additionally includes body mass index but does not include cholesterol-related variables.

We used the “AHAprevent” package to calculate the sex-specific CVD risk estimates across different combinations of ages and risk factor levels with the 10-year and 30-year PREVENT equations [11]. We selected the following variables to vary: age, HDL-C, TC, SBP, eGFR, diabetes, and anti-hypertensive or statin treatment status for the base equations; and UACR, HbA1c, and SDI for the expanded equations. Next, we calculated the ages at which clinically meaningful risk thresholds would be met, including when 10-year PREVENT-CVD risk estimates would exceed 7.5 % (recommended in the AHA/ACC 2025 High Blood Pressure Guidelines) and 20 % (defined as the risk equivalent for Stage 3 CKM Syndrome) [6,7,12]. Secondary analyses were conducted with the expanded PREVENT-CVD equations including the optional predictors (UACR, HbA1c, and SDI). All analyses were performed in R statistical software version 4.2.3 (R Foundation for Statistical Computing; Vienna, Austria).

### 2.2 Risk calculation varying a single predictor

First, we varied age in single year intervals from 30–79 years for 10-year CVD risk estimates and 30–59 years for 30-year CVD risk estimates for a hypothetical female or male with average risk factor levels based on overall population estimates from the National Health and Nutrition Examination Survey (NHANES) 2011-March 2020 data (**Supplemental Table 1**) [13]. For secondary analyses with the expanded equations, UACR was defined as the sex-specific mean values from the PREVENT derivation cohorts and SDI was defined as the 50th percentile (e.g., 5th decile) to reflect the median population estimate [4]. For categorical predictors, we selected no diabetes, non-smoking, and no anti-hypertensive or statin treatment to represent optimal and the normative values in the population. We varied individual risk factor levels across clinically meaningful ranges as selected in the development of the PREVENT-CVD equations for TC (130–320 mg/dL), HDL-C (20–100 mg/dL), SBP (90–200 mmHg), eGFR (15–120 ml/min/1.73m^2^), UACR (0.1–1000 mg/g), HbA1c (6.5–15 % in an individual with diabetes), and SDI deciles (1–10) [4]. We also compared predicted risk with and without anti-hypertensive or statin treatment while varying respective risk factor levels (SBP or TC).

### 2.3 Risk calculation varying multiple predictors

To examine how different combinations of risk factors influence absolute CVD risk estimates, we varied multiple risk factor levels simultaneously (e.g., with or without diabetes, or less or more advanced chronic kidney disease [CKD]) at a single age of 50 years for a hypothetical female or male. Specifically, we compared predicted risks with or without diabetes with either Stage 2 CKD (eGFR 75 mL/min/1.73m^2^) or Stage 3 CKD (eGFR 45 mL/min/1.73m^2^) using the base PREVENT equations. We selected these definitions of CKD as clinically meaningful as they represent the midpoint eGFR of these CKD stages based on the KDIGO 2024 clinical practice guidelines [6,14]. For the expanded PREVENT equations with optional predictors, we varied UACR across different CKD stages with or without diabetes. Based on KDIGO 2024 clinical practice guidelines, we examined UACR values of 15 mg/g (“normal to mildly increased”), 150 mg/g (“moderately increased”), and 500 mg/g (“severely increased”) with Stage 2 CKD (eGFR 75 mL/min/1.73m^2^), Stage 3 CKD (eGFR 45 mL/min/1.73m^2^), or Stage 4 CKD (eGFR 23 mL/min/1.73m^2^) in a hypothetical individual with or without diabetes [6,14]. All other risk factors were held at average population levels in these analyses.

### 2.4 Comparisons to prior risk assessment tools

Lastly, we compared 10-year sex-specific risk estimates with the PREVENT base equations to prior risk assessment tools developed in the United States using average risk factor levels from age 30 to 79 years. We compared PREVENT-ASCVD risk estimates with the Pooled Cohort Equations (PCEs), and PREVENT-HF risk estimates with the Pooled Cohort Equations to Prevent Heart Failure (PCP-HF) [15,16].

## 3 Results

### 3.1 Ten-year risk estimates with the PREVENT base equations

**Fig. 1:**
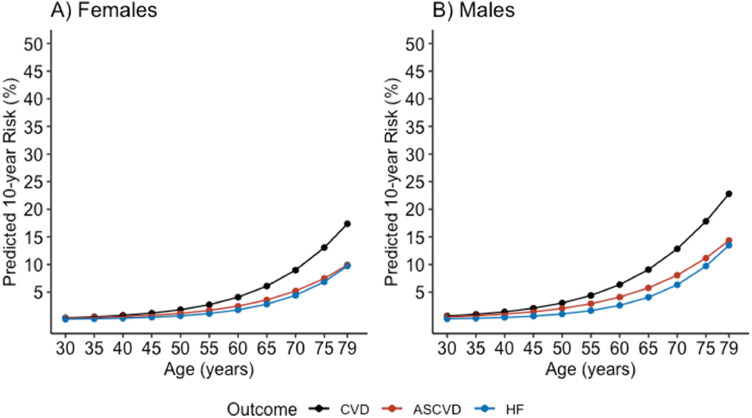
**Ten-year cardiovascular disease risk estimates with the American Heart Association PREVENT™ base equations varying age.** Ten-year risk estimates for total CVD, atherosclerotic CVD [ASCVD], and heart failure [HF] with the PREVENT base equations in a hypothetical female (**A**) or male (**B**) from 30 to 79 years of age with average population-based values, no diabetes or smoking, and no anti-hypertensive or statin medication.

The sex-specific 10-year risk estimates from the PREVENT-CVD base equations from age 30 to 79 years for a hypothetical female or male with average population-based risk factor levels are shown in Fig. 1**.** Predicted risk of CVD was higher with older age, and higher than the risks for individual CVD subtypes (ASCVD, HF) at any given age. For females, 10-year CVD risk ranged from 0.3 % at age 30 years to 17.4 % at age 79 years, and for males from 0.7 % at age 30 years to 22.8 % at age 79 years. The ages at which different levels of CVD risk would be met with average risk factor levels are presented in Table 1. For example, the 7.5 % threshold (recommended for the initiation of medication therapy in the 2025 High Blood Pressure Guidelines) would be met with average risk factor levels at age 68 years for a hypothetical female and at age 63 years for a hypothetical male (**Central Illustration**). The ages at which different levels of 10-year ASCVD or HF risks would be met with average risk factor levels are shown in **Supplemental Table 2**. When compared with prior risk assessment tools, differences in 10-year predicted risk widened with older age for PREVENT-ASCVD versus the PCEs (**Supplemental Figure 1**), and PREVENT-HF versus the PCP-HF (**Supplemental Figure 2**).

**Table 1:** Ages at which predicted 10-year risk with PREVENT-CVD would exceed clinically meaningful thresholds.

PREVENT-CVD 10-Year Risk	Female	Male
0.5 %	35 years	<30 years
1 %	43 years	36 years
3 %	57 years	50 years
5 %	63 years	57 years
7.5 %	68 years	63 years
10 %	72 years	67 years
15 %	77 years	73 years
20 %	>79 years	77 years

Values represent the approximate age that a hypothetical female or male with risk factors at average population levels (**Supplemental Table 1**), no diabetes or smoking, and no anti-hypertensive or statin medication would reach each risk threshold based on 10-year total CVD risk with the PREVENT base equations.

### 3.2 Ten-year risk estimates with the PREVENT-CVD base equations, varying single predictors

**Fig. 2:**
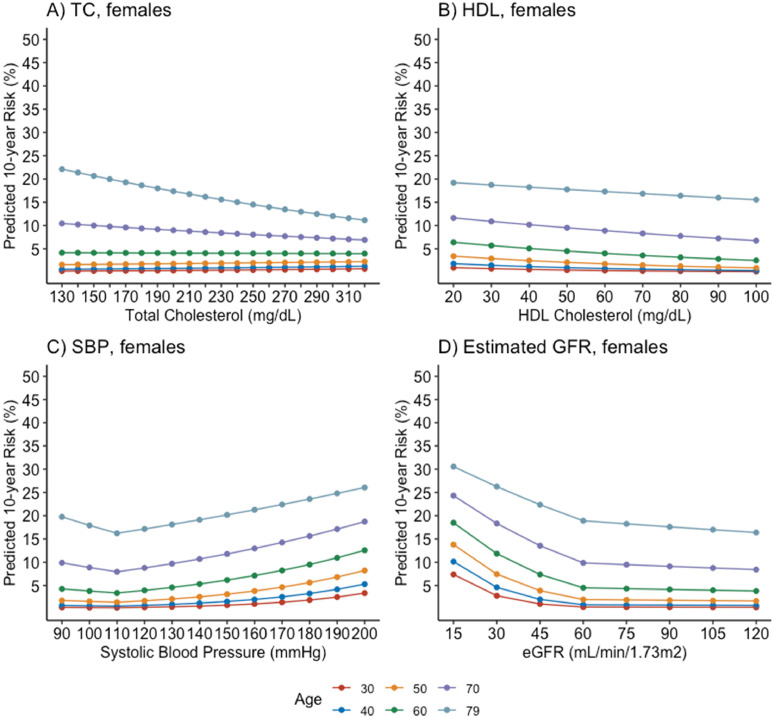
**Ten-year cardiovascular disease risk estimates with the American Heart Association PREVENT™ base equations varying individual risk factor levels, females.** Ten-year risk estimates for total CVD in a hypothetical female for untreated total cholesterol [TC] (**A**), untreated high density lipoprotein cholesterol [HDL-C] (**B**), untreated systolic blood pressure [SBP] (**C**), and estimated glomerular filtration rate [eGFR] (**D**) across a range of clinically meaningful values at selected ages. All other risk factors held at average overall population-based values with no diabetes or smoking. Abbreviations as in Supplemental Table 1.

**Fig. 3:**
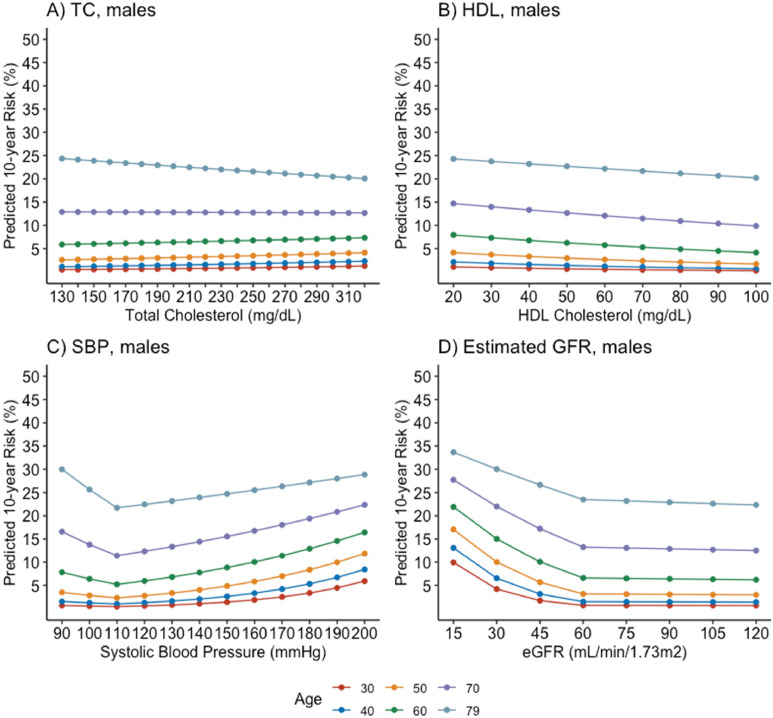
**Ten-year cardiovascular disease risk estimates with the American Heart Association PREVENT™ base equations varying individual risk factor levels, males.** Ten-year risk estimates for total CVD in a hypothetical male for untreated TC (**A**), untreated HDL-C (**B**), untreated SBP (**C**), and eGFR (**D**) across a range of clinically meaningful values at selected ages. All other risk factors held at average overall population-based values with no diabetes or smoking. Abbreviations as in Fig. 2.

Predicted 10-year PREVENT-CVD risk varied significantly across the range of TC, HDL-C, SBP, and eGFR values for females (Fig. 2) and males (Fig. 3). There were modest changes for risk based on TC and more pronounced linear variation for risk with SBP levels higher than 110 mmHg. There was a curvilinear pattern with varying eGFR levels. With all other risk factors held constant at average levels, predicted risk for a hypothetical 50-year-old female across the full range of risk factor levels ranged from 1.6 % to 2.2 % for TC (130–320 mg/dL), 0.9 % to 3.4 % for HDL-C (20–100 mg/dL), 1.4 % to 8.2 % for SBP (90–200 mmHg), and 1.7 % to 13.8 % for eGFR (15–120 ml/min/1.73m^2^). Similar patterns were observed for a hypothetical 50-year-old male but with higher levels of predicted risk (2.6 % to 4.1 % for TC, 1.7 % to 4.1 % for HDL-C, 2.3 % to 11.9 % for SBP, and 3.0 % to 17.1 % for eGFR). The impact of anti-hypertensive treatment or statin treatment across the range of SBP or TC values, respectively, is shown in **Supplemental Figure 3.**

### 3.3 Ten-year risk estimates with the PREVENT-CVD base equations, varying multiple predictors

**Fig. 4:**
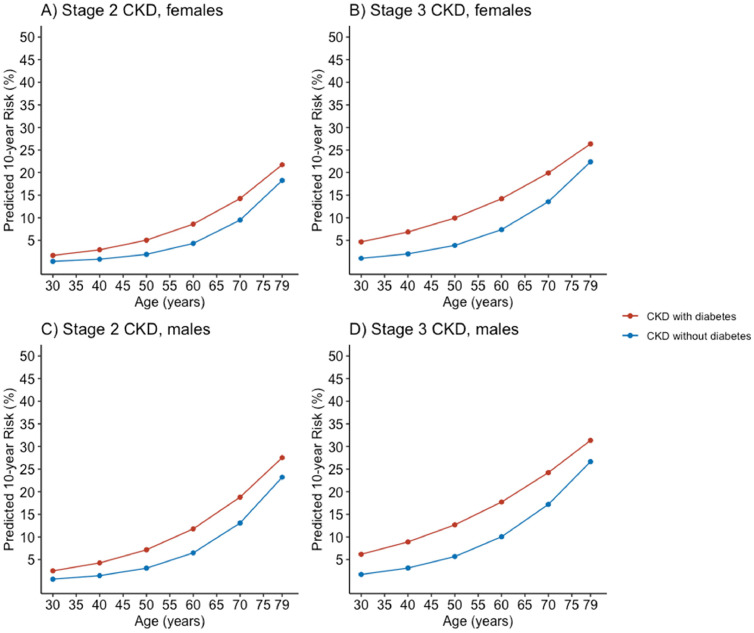
**Ten-year cardiovascular disease risk estimates with the American Heart Association PREVENT™ base equations varying diabetes and chronic kidney disease status.** Ten-year risk estimates for total CVD in a hypothetical female (top panels) or male (bottom panels) from 30 to 79 years of age with Stage 2 chronic kidney disease [CKD] (**A, C**) or Stage 3 CKD (**B, D**) while varying diabetes status. All other risk factors held at average overall population-based values with no smoking and no anti-hypertensive or statin medication.

Both diabetes and CKD were associated with higher CVD risk estimates (Fig. 4). In a hypothetical female with all other risk factors at average levels, the age at which predicted risk would exceed the 7.5 % risk threshold was 67 years if Stage 2 CKD was present without diabetes, 61 years if Stage 3 CKD was present without diabetes, 58 years if Stage 2 CKD was present with diabetes, and 43 years if Stage 3 CKD was present with diabetes, with similar patterns but at younger ages for a hypothetical male (Stage 2 CKD without diabetes: 63 years; Stage 3 CKD without diabetes: 55 years; Stage 2 CKD with diabetes: 51 years; Stage 3 CKD with diabetes: 36 years). In a hypothetical individual with Stage 3 CKD with all other risk factors at average levels, predicted risk would exceed the 20 % threshold (which defines Stage 3 CKM syndrome) for a female at 77 years without diabetes and 71 years with diabetes, and for a male at 73 years without diabetes and at 64 years with diabetes.

### 3.4 Ten-year risk estimates with the PREVENT-CVD expanded equations, varying single predictors

**Fig. 5:**
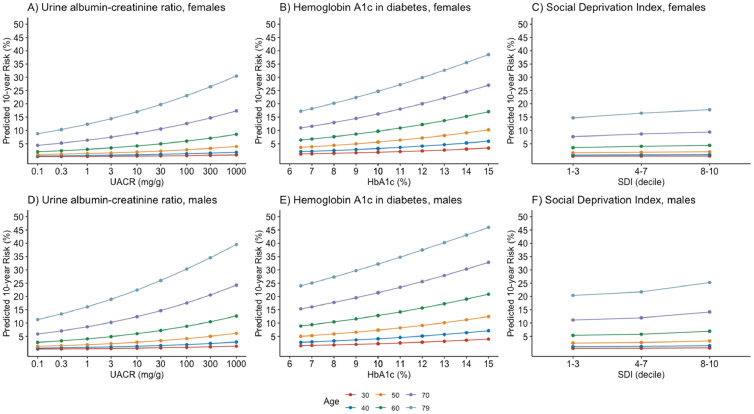
**Ten-year cardiovascular disease risk estimates with the American Heart Association PREVENT™ expanded equations varying individual risk factor levels.** Ten-year risk estimates for total CVD in a hypothetical female (**A-C**) or male (**D-F**) for urine albumin-creatinine ratio [UACR] (**A, D**), hemoglobin A1c [HbA1c] in diabetes (**B, E**), and Social Deprivation Index [SDI] (**C, F**) across a range of clinically meaningful values at selected ages. All other risk factors held at average overall population-based values with no anti-hypertensive or statin medication, and no smoking or diabetes (unless otherwise indicated). UACR is presented on a logarithmic scale.

Fig. 5 displays predicted 10-year CVD risks with the PREVENT expanded equations when varying the optional predictors of UACR, HbA1c, and SDI with all other risk factors held at average population-based levels. There was a curvilinear pattern with varying UACR and HbA1c levels, and linear variation with higher SDI. At age 50 years and with all other risk factors held constant at average levels, predicted CVD risk for a hypothetical female across the full range of risk factor levels ranged from 0.9 % to 4.0 % for UACR (0–1000 mg/g), 3.6 % to 10.2 % for HbA1c in diabetes (6.5–15 %), and 1.6 % to 2.0 % for SDI (deciles 1–10), with similar patterns for a hypothetical male but with higher levels of predicted risk (1.3 % to 6.2 % for UACR, 5.0 % to 12.5 % for HbA1c in diabetes, and 2.5 % to 3.3 % for SDI).

### 3.5 Ten-Year Risk Estimates with the PREVENT-CVD Expanded Equations, Varying Multiple Predictors

**Fig. 6:**
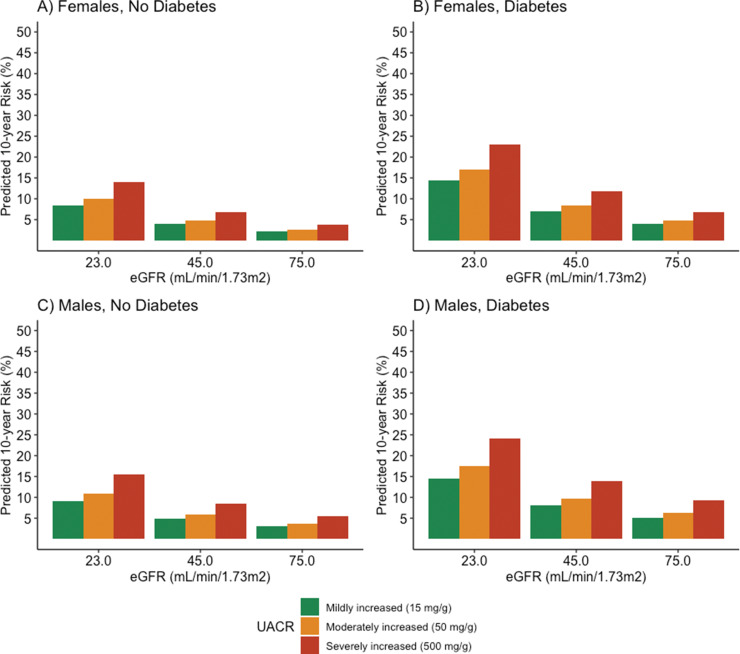
**Ten-year cardiovascular disease risk estimates with the American Heart Association PREVENT™ expanded equations varying measures of kidney health and diabetes status.** Ten-year risk estimates for total CVD in a hypothetical 50-year-old female (**A-B**) or male (**C-D**) with or without diabetes for selected UACR values (15, 50, or 500 mg/g) and eGFR values (23, 45, or 75 mL/min/1.73m^2^). All other risk factors held at average overall population-based values with no diabetes or smoking, and no anti-hypertensive or statin medication. Abbreviations as in Fig. 2, Fig. 5.

Given both eGFR and UACR are independent predictors of CVD risk and are used in combination for assessment of kidney health, we varied eGFR with UACR simultaneously at age 50 years with all other risk factors held at average population levels (Fig. 6). For example, in a hypothetical 50-year-old female with no diabetes and otherwise average risk factor levels, predicted CVD risk ranged depending on the UACR level from 2.2 % to 3.8 % with Stage 2 CKD, from 3.9 % to 6.8 % with Stage 3 CKD, and from 8.4 % to 14.0 % with Stage 4 CKD. If diabetes was present in this hypothetical female, predicted CVD risk ranged depending on the UACR level from 3.9 % to 6.8 % with Stage 2 CKD, from 6.9 % to 11.7 % with Stage 3 CKD, and from 14.4 % to 23.0 % with Stage 4 CKD.

### 3.6 Thirty-year risk estimates with the PREVENT equations

Similar patterns for risk estimates were observed with the 30-year PREVENT equations across all risk factor variations, but with higher absolute risk estimates for 30-year risk when compared with corresponding 10-year risk (**Supplemental Figures 4–10**). With average population-based risk factor levels, 30-year risk estimates from the PREVENT-CVD base equations from age 30 to 59 years ranged from 2.5 % to 20.5 % for a hypothetical female and from 4.8 % to 26.5 % for a hypothetical male, and the ages at which 30-year risk estimates would exceed various risk thresholds are shown in **Supplemental Tables 3–4**.

## 4 Discussion

In this systematic examination of the PREVENT equations, we demonstrate several important findings. First, even at younger ages, there is substantial heterogeneity in CVD risk estimates, particularly based on predictors relevant to CKM health (eGFR, UACR, HbA1c, SDI) or when considering a longer time horizon (e.g., using 30-year risk estimates). This provides clinicians with the tools to discuss and initiate risk-based prevention strategies earlier in the life course as displayed in the **Central Illustration**. Second, the expanded outcome of total CVD enables a more comprehensive risk estimate of CVD that is higher than that estimated for ASCVD or HF alone. Third, we highlight age and risk factor combinations that exceed the clinically relevant threshold recommended in the 2025 AHA/ACC High BP Guideline (10-year PREVENT-CVD ≥7.5 %) and that define Stage 3 CKM Syndrome (10-year PREVENT-CVD ≥20 %) to support implementation and interpretation among clinicians and patients.

The PREVENT equations newly provide risk estimates for individuals beginning at age 30 years and incorporate predictors of kidney function that refine risk estimates. For example, 10-year PREVENT-CVD risk estimates for a hypothetical male with diabetes and Stage 3 CKD would already exceed the 7.5 % threshold before 40 years of age [12]. This distinction was not present in prior risk models as they did not include eGFR or account for risk associated with kidney disease [8]. Moreover, the inclusion of optional predictors with UACR and HbA1c demonstrates how the confluence of adverse kidney and metabolic health led to substantial heterogeneity in predicted risk. The inclusion of these optional predictors offers several benefits beyond personalizing risk, including nudging clinicians to screen for these markers when indicated and optimize treatment. Given the substantial under-utilization of evidence-based screening with UACR, even when indicated, and the emergence of evidence-based therapies with kidney protective benefits (e.g., sodium-glucose cotransporter-2 inhibitors, glucagon-like peptide 1 receptor agonists, and non-steroidal mineralocorticoid receptor antagonists), the PREVENT equations may support the promotion of CKM health through the identification and treatment of high-risk individuals (i.e., Stage 3 CKM) [6,7,17]. Future research is needed to evaluate the cost-effectiveness of these novel CKM therapies based on the 20 % threshold for 10-year PREVENT-CVD risk that corresponds to Stage 3 CKM.

The integration of zip code-level SDI represents a first step at integrating social exposures in risk prediction. The present analysis demonstrated that estimated risk varied for a hypothetical individual with the same risk factor levels across deciles of SDI. This underscores the importance of assessing and addressing social needs or non-medical factors that contribute to the risk of CVD. The value of incorporating social determinants of health (SDoH) has also been demonstrated in several risk prediction algorithms like the United Kingdom’s QRISK score for CVD [18], the Australian CVD risk calculator [19], and when added to the Pooled Cohort Equations [20]. Given the upstream impacts of SDoH on CVD risk and outcomes, SDI is a valuable metric in PREVENT risk calculations, and can prompt clinicians to consider SDoH in CVD risk assessment [5,21]. However, SDI only accounts for area-level exposures at the zip-code level and does not address individual-level social risk and resilience. There may also be significant heterogeneity in social adversity within one zip code. Therefore, the role of individual-level social factors, and whether accounting for such factors enhances risk prediction, should be evaluated in future iterations of risk models.

As in prior risk prediction tools, age is a dominant driver of absolute risk estimates with PREVENT, both due to the main coefficient for age and age-specific risk factor level coefficients [8]. However, even among those with optimal risk factor levels, or lower absolute risk due to younger age, prior studies have shown that many individuals in the United States who are at low short-term risk are at high long-term risk for CVD [[22], [23], [24]]. Therefore, 30-year risk estimates offer the opportunity to target prevention efforts with lifestyle or pharmacotherapy earlier in the life course. This is especially critical for younger adults, given the growing burden of risk factors such as diabetes, obesity, and hypertension among this age group [25]. Importantly, our findings highlight that 30-year risk is not simply rescaled 10-year risk given the influence of age- and risk factor-specific coefficients in each model, as demonstrated when comparing 10-year and 30-year risks across clinical risk factor profiles.

Some of the predicted risk outputs also highlight some observations that may appear paradoxical and are among the most frequently asked questions by clinicians and patients. First, anti-hypertensive treatment was associated with a higher risk estimate for the same BP level, and is consistent with what was observed with prior risk scores [[8], [9], [10]]. This reflects the greater underlying risk in someone on anti-hypertensive treatment due to a longer burden and/or severity of the risk factor compared to a different individual with the same risk factor levels but not receiving any treatment [26,27]. This also highlights that the PREVENT equations cannot be used to inform change in predicted risk after initiating treatment within a given individual, which requires inputs from randomized clinical trials to estimate dynamic risk with initiation of preventive strategies, as was implemented in the development of the Million Hearts Model [26]. Second, lower levels of total cholesterol (treated or untreated) were associated with a higher total CVD risk estimate after 70 years of age, which may be due to survival bias or reverse causation. Third, SBP lower than 110 mm Hg was associated with a higher risk of CVD, which is also an example of reverse causation. Whereas these risk factor levels may represent optimal health among younger adults, a low cholesterol or SBP value at an elder age may indicate underlying multimorbidity, physical frailty, or deteriorating nutritional status [[28], [29], [30]].

### 4.1 Limitations

A few limitations of these analyses should be noted. First, these are hypothetical scenarios and many risk factors are often correlated and cluster together. Therefore, some proposed combinations may be unlikely to be observed in clinical practice. Second, values outside the range defined in PREVENT development were not examined, although they may be encountered in clinical practice. To mitigate these concerns and support the generalizability of the findings, we used average risk factor levels based on data from a nationally representative sample (NHANES). Third, risk factors are also impacted by aging (including SBP and eGFR), and we used fixed averages across the age range examined in these hypothetical scenarios. Fourth, while long-term risk estimation may be valuable for targeted prevention at earlier ages, risk thresholds for 30-year risk estimates have not yet been established, and the interpretation of these estimates is challenging and may be supported by alternate approaches (e.g., percentiles) [31]. Finally, while open source software code for implementing the PREVENT equations is available through the AHA, integration into electronic medical records nationally for the automatic calculation of PREVENT risk estimates can further support clinical implementation [11].

## 5 Conclusion

The PREVENT equations offer clinicians and patients a set of outcome-specific risk estimates (PREVENT-CVD, PREVENT-ASCVD, and PREVENT-HF) for 10-year and 30-year time horizons. Optional predictors with UACR, HbA1c, and SDI enable more personalized risk assessment for kidney, metabolic, and social risk. This systematic examination supports communication of predicted risks for a variety of clinical scenarios and identifies the ages at which clinically meaningful risk thresholds may be met. These findings can also guide help risk communication strategies and optimize prevention for patients across the life course.

## Disclosures and funding

Donald M. Lloyd-Jones reports employment with American Heart Association. Josef Coresh reports a relationship with Healthy.io Ltd that includes consulting or advisory, and equity or stocks. All other authors have no known competing financial interests or personal relationships that could have appeared to influence the work reported in this paper.

## Author agreement

All authors have agreed to this manuscript, have participated in the preparation of the manuscript, and meet criteria for authorship. The manuscript represents original research and is not under consideration for publication elsewhere nor published elsewhere in whole or in part.

## CRediT authorship contribution statement

**Vaishnavi Krishnan:** Writing – original draft, Visualization, Investigation, Formal analysis, Conceptualization. **Xiaoning Huang:** Writing – review & editing, Software, Methodology, Formal analysis. **Chiadi E. Ndumele:** Writing – review & editing, Conceptualization. **Janani Rangaswami:** Writing – review & editing, Conceptualization. **Josef Coresh:** Writing – review & editing, Conceptualization. **Amanda M. Perak:** Writing – review & editing, Methodology, Conceptualization. **Nilay S. Shah:** Writing – review & editing, Supervision. **Donald M. Lloyd-Jones:** Writing – review & editing, Supervision, Methodology, Conceptualization. **Sadiya S. Khan:** Writing – review & editing, Supervision, Project administration, Conceptualization.

## Declaration of competing interest

The authors declare the following financial interests/personal relationships which may be considered as potential competing interests: Donald M. Lloyd-Jones reports a relationship with American Heart Association Inc that includes: employment. Josef Coresh reports a relationship with Healthy.io Ltd that includes: consulting or advisory and equity or stocks. If there are other authors, they declare that they have no known competing financial interests or personal relationships that could have appeared to influence the work reported in this paper.

## Appendix Supplementary materials

Image, application 1
