# Supplementary material for: Systematic examination of the PREVENT equations for cardiovascular disease risk
Source: Am J Prev Cardiol. 2026 Mar 13;27:101502. doi: 10.1016/j.ajpc.2026.101502 (PMC13261269; doi:10.1016/j.ajpc.2026.101502)
Supplement: Supplementary file 1 [file mmc1.docx]

**ONLINE ONLY SUPPLEMENTAL MATERIALS**

**Supplemental Table 1: Average population levels for cardiovascular disease risk factors included in PREVENT^TM^ risk estimates.** Values are mean (SE) or median (IQR). Average population values are derived from 2011–March 2020 National Health and Nutrition Examination Survey data^1^ for total cholesterol [TC], high density lipoprotein cholesterol [HDL-C], systolic blood pressure [SBP], body mass index [BMI], estimated glomerular filtration rate [eGFR] (calculated using the 2021 CKD-EPI creatinine equations^2^), and hemoglobin A1c [HbA1c]; and from the PREVENT derivation cohort^3^ for urine albumin-creatinine ratio [UACR].

|  | Males | Females |
| --- | --- | --- |
| Total cholesterol (TC, mg/dL) | 195.7 (0.7) | 200.0 (0.7) |
| High-density lipoprotein cholesterol (HDL-C, mg/dL) | 47.8 (0.3) | 58.6 (0.3) |
| Systolic blood pressure (SBP, mmHg) | 124.9 (0.3) | 122.2 (0.3) |
| Body mass index (BMI, kg/m^2^) | 29.4 (0.1) | 29.7 (0.2) |
| Estimated glomerular filtration rate (eGFR, ml/min/1.73m^2^) | 95.3 (82.1-106.2) | 96.0 (82.0-108.0) |
| Hemoglobin A1c (HbA1c, %) | 5.7 (0.0) | 5.7 (0.0) |
| Urine albumin-creatinine ratio (UACR, mg/g) | 8 (8–12) | 8 (8–12) |

**Supplemental Table 2: Age at which predicted 10-year risks with PREVENT-ASCVD or PREVENT-HF would exceed clinically meaningful thresholds.** Values represent the approximate age that a hypothetical female or male with risk factors at average population levels, no diabetes or smoking, and no anti-hypertensive or statin medication would reach each risk threshold based on 10-year atherosclerotic CVD [ASCVD] and heart failure [HF] risks with the PREVENT base equations.

|  | **ASCVD** | | **HF** | |
| --- | --- | --- | --- | --- |
| **Risk Threshold** | **Female** | **Male** | **Female** | **Male** |
| 0.5% | 40 years | 31 years | 47 years | 42 years |
| 1% | 49 years | 40 years | 54 years | 50 years |
| 3% | 63 years | 56 years | 66 years | 62 years |
| 5% | 70 years | 63 years | 72 years | 68 years |
| 7.5% | 76 years | 69 years | 76 years | 72 years |
| 10% | >79 years | 74 years | >79 years | 76 years |
| 15% | >79 years | >79 years | >79 years | >79 years |
| 20% | >79 years | >79 years | >79 years | >79 years |
| 25% | >79 years | >79 years | >79 years | >79 years |

**Supplemental Table 3: Age at which predicted 30-year risk with PREVENT-CVD would exceed various thresholds.** Values represent the approximate age that a hypothetical female or male with risk factors at average population levels, no diabetes or smoking, and no anti-hypertensive or statin medication would reach each risk threshold based on 30-year total CVD risk using the PREVENT base equations.

| **Risk Threshold** | **Female** | **Male** |
| --- | --- | --- |
| 0.5% | <30 years | <30 years |
| 1% | <30 years | <30 years |
| 3% | 32 years | <30 years |
| 5% | 38 years | 31 years |
| 7.5% | 43 years | 36 years |
| 10% | 46 years | 40 years |
| 15% | 53 years | 46 years |
| 20% | 59 years | 52 years |
| 25% | >59 years | 58 years |

**Supplemental Table 4: Age at which predicted 30-year risks with PREVENT-ASCVD or PREVENT-HF would exceed various thresholds.** Values represent the approximate age that a hypothetical female or male with risk factors at average population levels, no diabetes or smoking, and no anti-hypertensive or statin medication would reach each risk threshold based on 30-year atherosclerotic CVD [ASCVD] and heart failure [HF] risks with the PREVENT base equations.

|  | **ASCVD** | | **HF** | |
| --- | --- | --- | --- | --- |
| **Risk Threshold** | **Female** | **Male** | **Female** | **Male** |
| 0.5% | <30 years | <30 years | <30 years | <30 years |
| 1% | <30 years | <30 years | <30 years | <30 years |
| 3% | 37 years | <30 years | 41 years | 37 years |
| 5% | 44 years | 35 years | 47 years | 42 years |
| 7.5% | 50 years | 41 years | 52 years | 48 years |
| 10% | 56 years | 46 years | 57 years | 52 years |
| 15% | >59 years | 56 years | >59 years | >59 years |
| 20% | >59 years | >59 years | >59 years | >59 years |
| 25% | >59 years | >59 years | >59 years | >59 years |

**Supplemental Figure 1: Comparison of American Heart Association PREVENT^TM^ base equations for 10-year atherosclerotic cardiovascular disease risk with the Pooled Cohort Equations.** Ten-year risk estimates using PREVENT base equations for atherosclerotic CVD [ASCVD] versus Pooled Cohort Equations [PCEs] in a hypothetical female **(A)** or male **(B)** from 30 to 79 years of age with risk factors at average population levels, no diabetes or smoking, and no current medications. Risk estimates for PCEs begin at 40 years of age in accordance with its development. Predictors in PCEs include age, sex, race, total cholesterol, high density lipoprotein cholesterol, systolic blood pressure, use of anti-hypertensive medication, smoking status, and diabetes status.

**
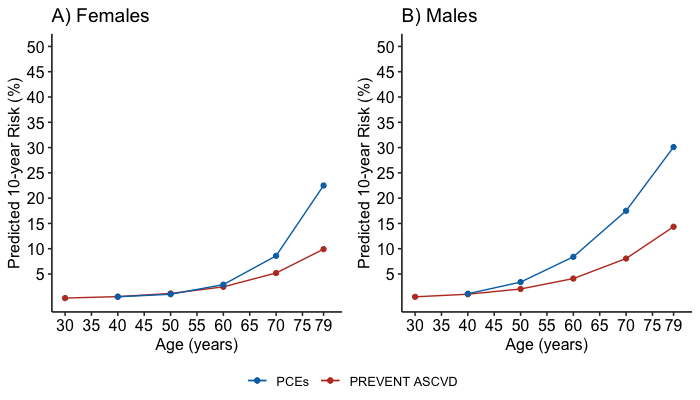
**

**Supplemental Figure 2: Comparison of American Heart Association PREVENT^TM^ base equations for 10-year heart failure risk with the Pooled Cohort Equations to Prevent Heart Failure.** Ten-year risk estimates using PREVENT base equations for heart failure [HF] versus Pooled Cohort Equations to Prevent Heart Failure [PCP-HF] in a hypothetical female **(A)** or male **(B)** from 30 to 79 years of age with risk factors at average population levels, no diabetes or smoking, and no current medications. Predictors in PCP-HF include age, sex, race, total cholesterol, high density lipoprotein cholesterol, systolic blood pressure, use of anti-hypertensive medication, smoking status, body mass index, fasting glucose, diabetes treatment, and QRS duration.


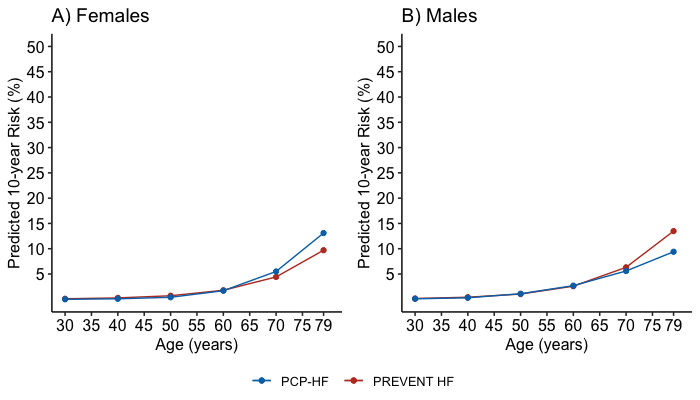


**Supplemental Figure 3: Impact of anti-hypertensive and statin treatment on 10-year cardiovascular disease risk with the American Heart Association PREVENT^TM^ base equations.** Ten-year risk estimates for total CVD in for treated TC **(A,C)** and treated SBP **(B, D)** in a female (top panels) and in a male (bottom panels) across a range of clinically meaningful values at selected ages. All other risk factors held at average overall population-based values with no diabetes or smoking. Abbreviations as in Supplemental Table 1.

**
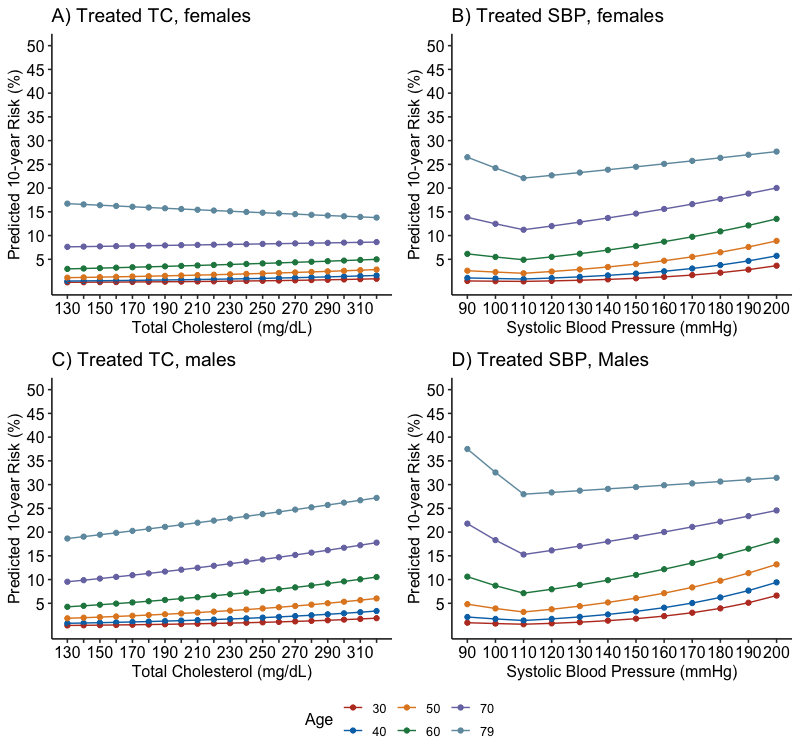
**

**Supplemental Figure 4: Thirty-year cardiovascular disease risk estimates with the American Heart Association PREVENT^TM^ base equations varying age.** Thirty-year risk estimates for total CVD, atherosclerotic CVD [ASCVD] , and heart failure [HF] with the PREVENT base equations in a hypothetical female **(A)** or male **(B)** from 30 to 59 years of age with risk factors at average population levels, no diabetes or smoking, and no anti-hypertensive or statin medication.

**
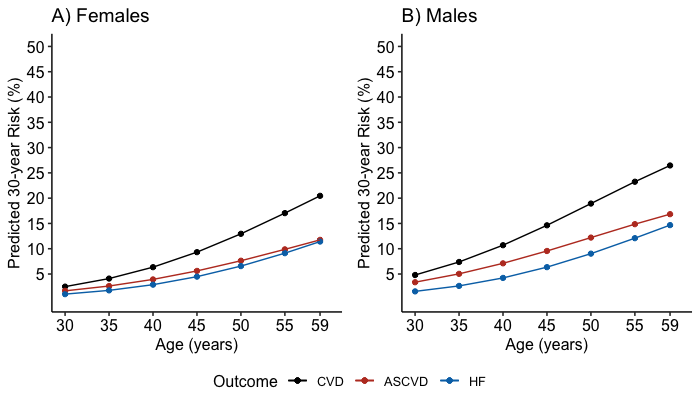
**

**Supplemental Figure 5: Thirty-year cardiovascular disease risk estimates with the American Heart Association PREVENT^TM^ base equations varying individual risk factor levels, females.** Thirty-year risk estimates for total CVD in a hypothetical female for untreated TC **(A)**, untreated HDL-C **(B)**, untreated SBP **(C)**, and eGFR **(D)** across a range of clinically meaningful values at selected ages. All other risk factors held at average overall population-based values with no diabetes or smoking. Abbreviations as in Supplemental Table 1.


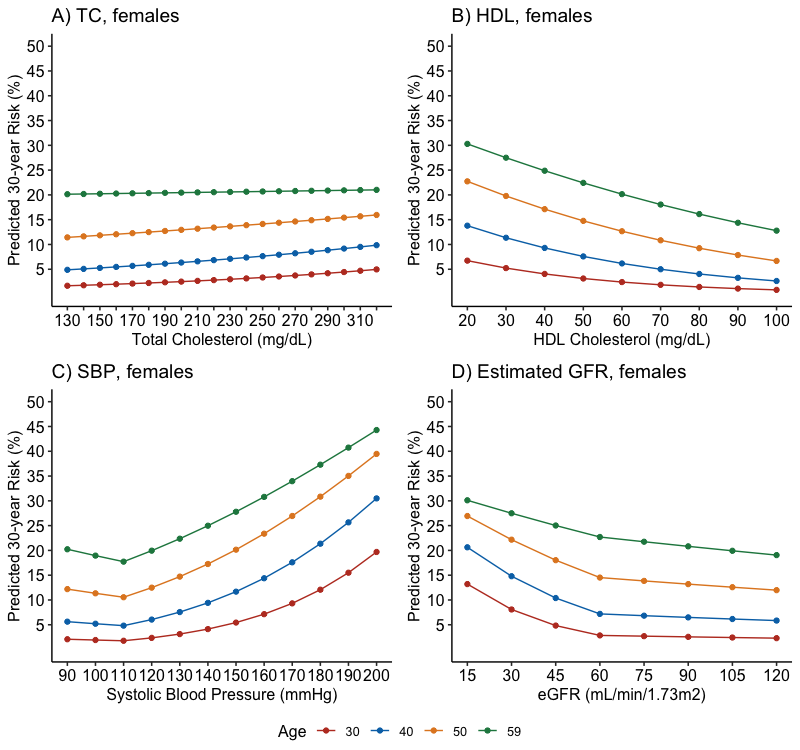


**Supplemental Figure 6: Thirty-year cardiovascular disease risk estimates with the American Heart Association PREVENT^TM^ base equations varying individual risk factor levels, males.** Thirty-year risk estimates for total CVD in a hypothetical male for untreated TC **(A)**, untreated HDL-C **(B)**, untreated SBP **(C)**, and eGFR **(D)** across a range of clinically meaningful values at selected ages. All other risk factors held at average overall population-based values with no diabetes or smoking. Abbreviations as in Supplemental Table 1.


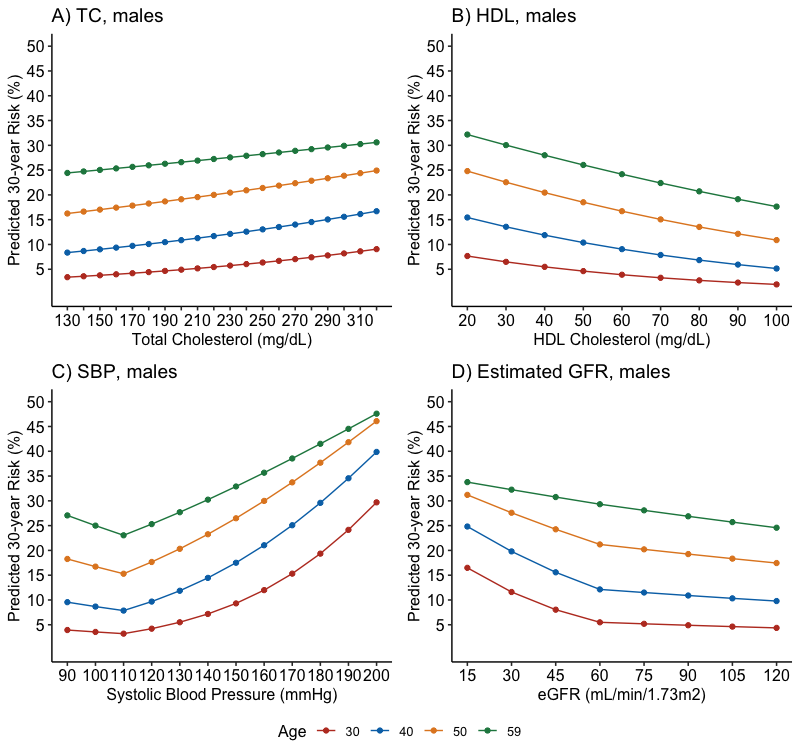


**Supplemental Figure 7: Impact of anti-hypertensive and statin treatment on 30-year cardiovascular disease risk with the American Heart Association PREVENT^TM^ base equations**. Thirty-year risk estimates for total CVD in for treated TC **(A,C)** and treated SBP **(B, D)** in a female (top panels) and in a male (bottom panels) across a range of clinically meaningful values at selected ages. All other risk factors held at average overall population-based values with no diabetes or smoking. Abbreviations as in Supplemental Table 1.


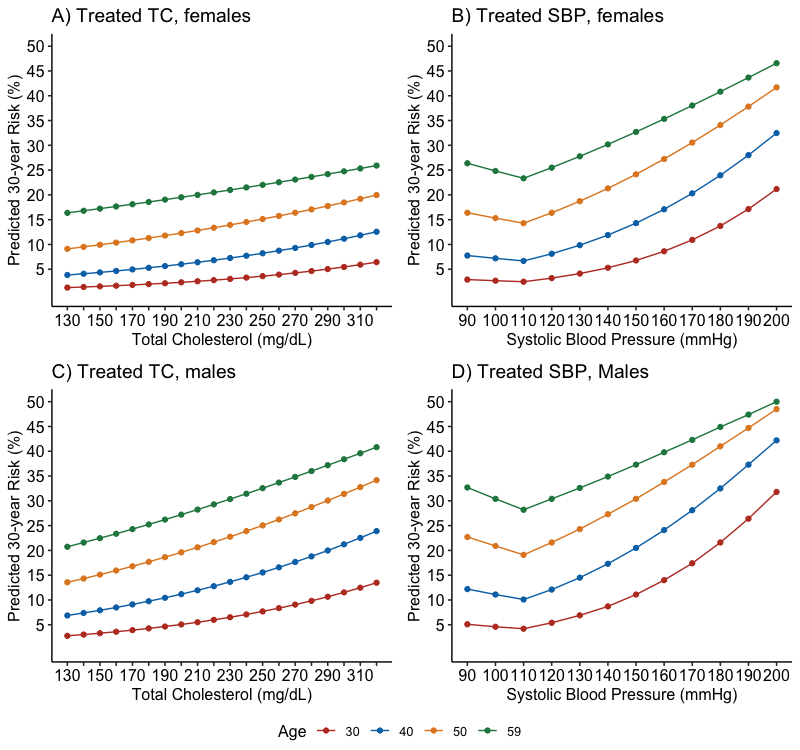


**Supplemental Figure 8: Thirty-year cardiovascular disease risk estimates with the American Heart Association PREVENT^TM^ base equations varying diabetes and chronic kidney disease status.** Thirty-year risk estimates for total CVD in a hypothetical female **(**top panels**)** or male **(**bottom panels**)** from 30 to 59 years of age with Stage 2 chronic kidney disease [CKD] (**A, C**) or Stage 3 CKD (**B, D**) while varying diabetes status. All other risk factors held at average overall population-based values with no smoking and no anti-hypertensive or statin medication.


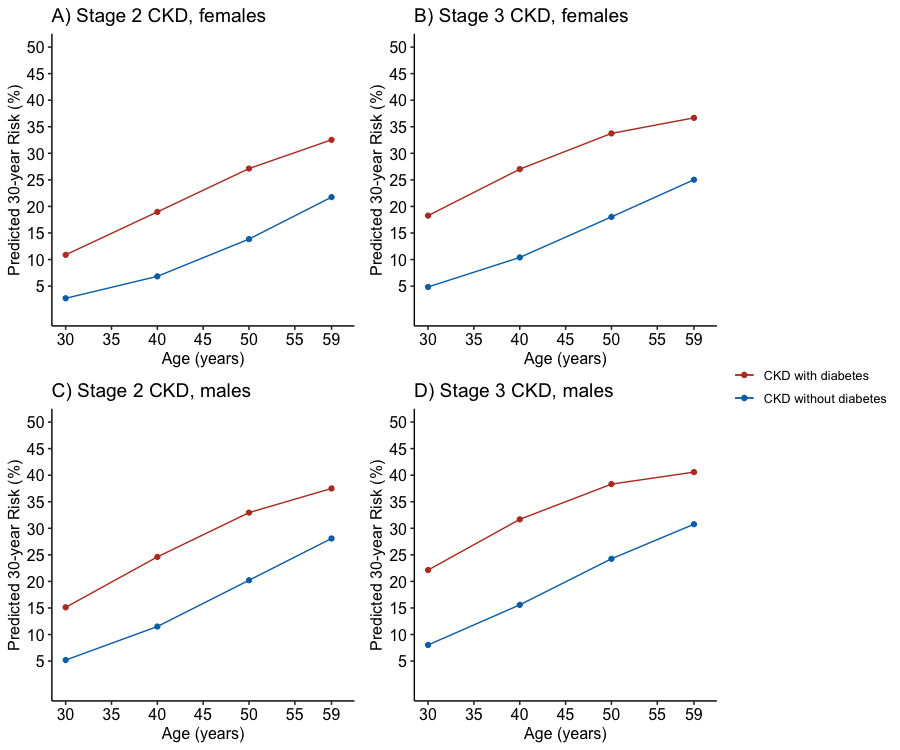


**Supplemental Figure 9: Thirty-year cardiovascular disease risk estimates with the American Heart Association PREVENT^TM^ expanded equations varying individual risk factor levels.** Thirty-year risk estimates for total CVD in a hypothetical female (**A-C**) or male (**D-F**) for urine albumin-creatinine ratio [UACR] **(A, D)**, hemoglobin A1c [HbA1c] in diabetes **(B, E)**, and Social Deprivation Index [SDI] **(C, F)** across a range of clinically meaningful values at selected ages. All other risk factors held at average overall population-based values with no anti-hypertensive or statin medication, and no smoking or diabetes (unless otherwise indicated). UACR is presented on a logarithmic scale.


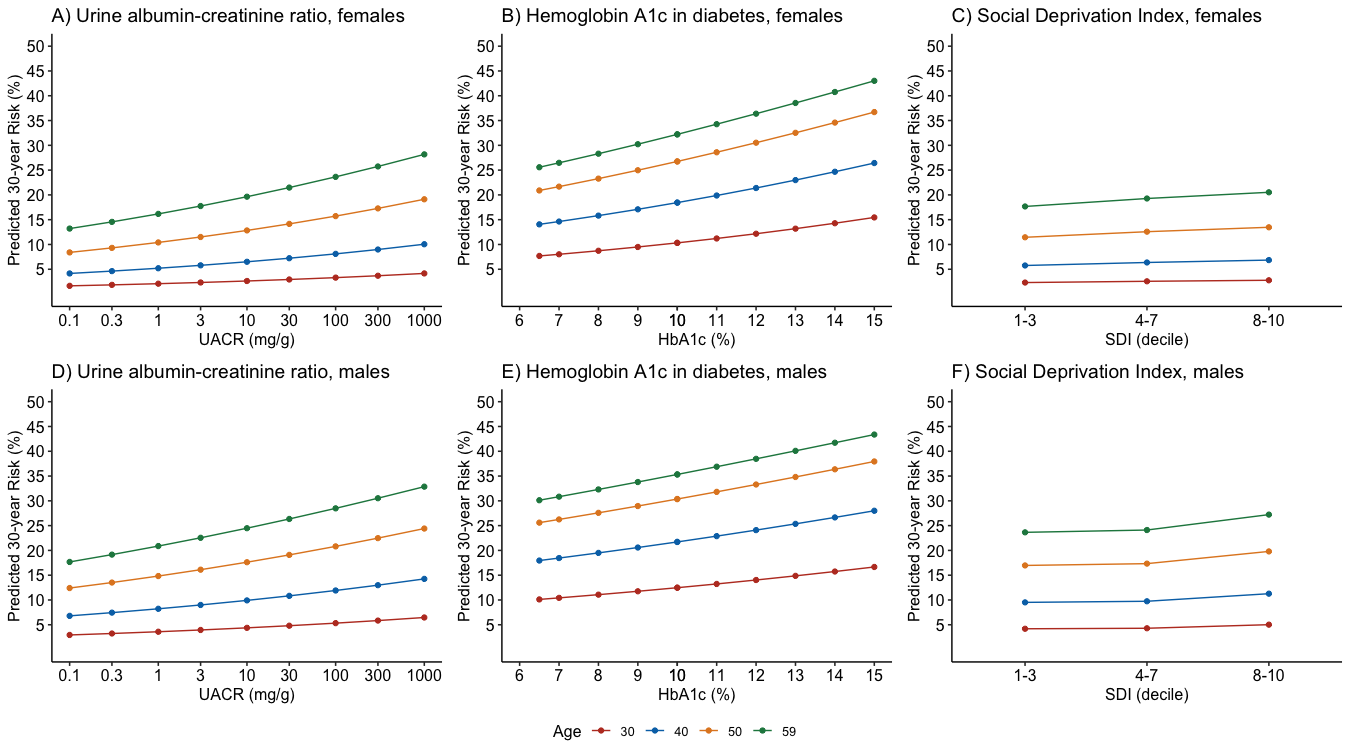


**Supplemental Figure 10: Thirty-year cardiovascular disease risk estimates with the American Heart Association PREVENT^TM^ expanded equations varying measures of kidney health and diabetes status.** Thirty-year risk estimates for total CVD in a hypothetical 50-year-old female **(A-B)** or male **(C-D)** with or without diabetes for selected urine albumin-creatinine ratio [UACR] values (15, 50, or 500 mg/g) and eGFR values (23, 45, or 75 mL/min/1.73m2). All other risk factors held at average overall population-based values with no diabetes or smoking, and no anti-hypertensive or statin medication. Abbreviations as in Supplemental Table 1.


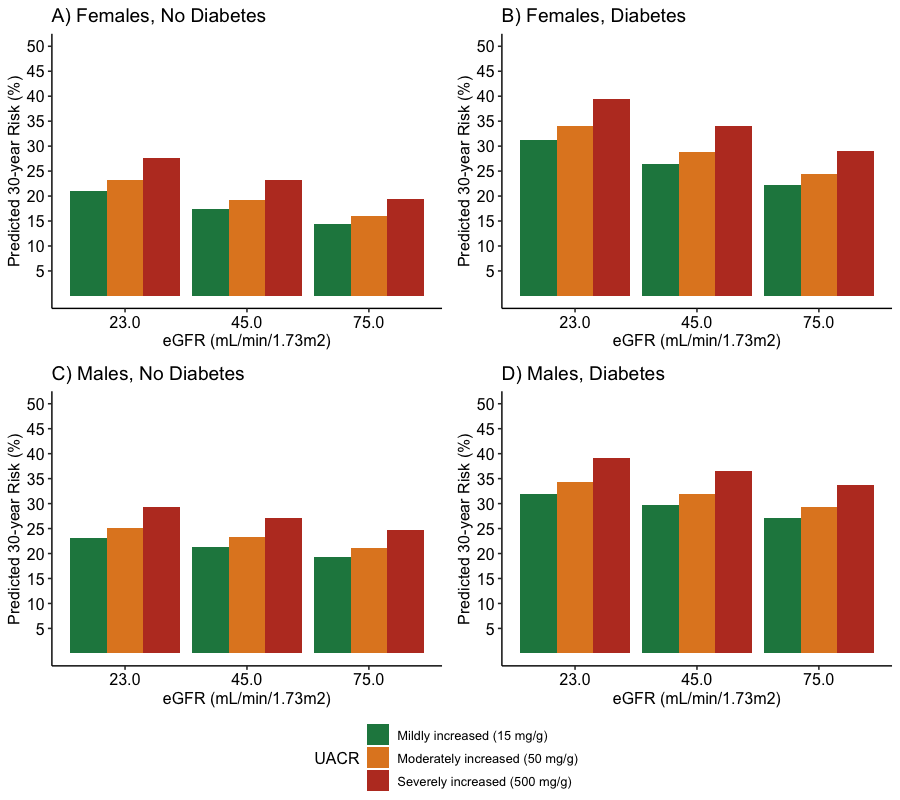


**SUPPLEMENTAL ONLY REFERENCES**

1. National Center for Health Statistics. National Health and Nutrition Examination Survey (NHANES). <https://wwwn.cdc.gov/nchs/nhanes/Default.aspx>

2. Inker LA, Eneanya ND, Coresh J, Tighiouart H, Wang D, Sang Y, Crews DC, Doria A, Estrella MM, Froissart M, et al. New Creatinine- and Cystatin C-Based Equations to Estimate GFR without Race. *N Engl J Med*. 2021;385:1737-1749. doi: 10.1056/NEJMoa2102953

3. Khan SS, Matsushita K, Sang Y, Ballew SH, Grams ME, Surapaneni A, Blaha MJ, Carson AP, Chang AR, Ciemins E, et al. Development and Validation of the American Heart Association's PREVENT Equations. *Circulation*. 2024;149:430-449. doi: 10.1161/CIRCULATIONAHA.123.067626
